# Effects of a 12-Week Group Circuit-Based Kinetotherapy Program on Muscle Strength, Segmental Trophicity, and Postural Balance in Children with Down Syndrome: An Exploratory Pre–Post Intervention Study with a Baseline Comparison Group

**DOI:** 10.3390/medsci14060666

**Published:** 2026-10-12

**Authors:** Andreea-Liana Bot (Rachisan), Bogdan Lucian, Madalina-Alexandra Moga, Diana-Flavia Jecan-Toader, Mihaela Sparchez

**Affiliations:** 1 Department 2, Faculty of Nursing and Health Sciences, University of Medicine and Pharmacy “Iuliu Hatieganu”, 400023 Cluj-Napoca, Romania; 2 Faculty of Nursing and Health Sciences, University of Medicine and Pharmacy “Iuliu Hatieganu”, 400023 Cluj-Napoca, Romania; 3 2nd Pediatric Discipline, Mother and Child Department, Faculty of Medicine, Iuliu Haţieganu University of Medicine and Pharmacy, 400023 Cluj-Napoca, Romania

**Keywords:** Down syndrome, trisomy 21, kinetotherapy, circuit training, pediatric rehabilitation, postural balance, Berg Balance Scale, hypotonia, group therapy, music therapy

## Abstract

**Background:** Children with Down syndrome (DS) commonly present hypotonia, ligamentous laxity, and impaired postural control, which delay gross motor milestones and limit functional independence. Group-based, music-supported circuit kinetotherapy has been proposed as a feasible and engaging strategy to address these deficits, but comparative evidence in pediatric samples remains limited. Patients and methods: A prospective, exploratory pre–post intervention study with a non-randomized baseline control group was conducted in 20 children with trisomy 21 (aged 4–12 years), allocated by feasibility of attendance (not randomized) to an experimental group (n = 10) that completed a 12-week, once-weekly, 60-min music-assisted dynamic circuit program (seven stations targeting coordination, proprioception, postural alignment, and functional reach) and a control group (n = 10) that received no structured intervention and was assessed at baseline only (no post-intervention follow-up data were collected for this group). Outcomes included segmental limb circumferences, manual muscle testing (F1–F5) of six muscle groups, and the Berg Balance Scale (BBS), assessed at baseline (T1) in both groups and after the intervention period (T2) in the experimental group only. **Results:** These within-group (pre–post) findings in the experimental group showed statistically significant increases in left arm circumference (contracted; *p* = 0.009, d = 0.25), left thigh circumference (*p* = 0.001, d = 0.16), right thigh circumference (*p* = 0.003, d = 0.17), iliopsoas manual muscle test scores (Wilcoxon *p* = 0.015; reported by Wilcoxon test only, as MMT is an ordinal scale), and total BBS score (from 41.4 ± 8.67 to 45.9 ± 7.85; t(9) = 6.40, *p* < 0.001, Wilcoxon *p* = 0.006, d = 0.54). Right arm circumference, triceps brachii, and elbow flexors showed positive but statistically non-significant trends (*p* = 0.081–0.346); pelvic fixation function and quadriceps femoris also did not reach significance, and the middle deltoid/supraspinatus grade showed a small, non-significant decrease after correction of a baseline data-entry error identified during peer review. No correction for multiple comparisons was applied, and post-intervention data were not available for the control group; these findings should therefore be interpreted as exploratory. **Conclusions:** A 12-week group circuit kinetotherapy program combined with musical stimulation was associated with measurable within-group gains in segmental limb circumference, hip-flexor strength, and functional postural balance in children with Down syndrome. Because post-intervention data were not available for the control group, these findings provide preliminary, exploratory evidence rather than confirmation of superiority over no intervention. Larger, randomized controlled trials with complete follow-up in both arms are warranted to confirm these findings.

## 1. Introduction

Down syndrome (DS), caused by trisomy of chromosome 21, is the most common autosomal chromosomal disorder and is associated with a well-characterized profile of neuro-motor impairment, including generalized muscular hypotonia, ligamentous laxity, and delayed acquisition of gross motor milestones such as independent sitting and walking [1,2]. These primary impairments frequently translate into secondary deficits in postural control, static and dynamic balance, and coordination, which persist beyond early childhood and constrain participation in school, play, and self-care activities [3,4,5]. Systematic reviews and meta-analyses have consistently reported that structured physical exercise programs improve balance and gross motor function in children with DS, although heterogeneity in intervention design, dosage, and outcome measures limits direct comparison across studies [6,7,8]. Group-based approaches that combine motor learning principles with social interaction are of particular interest in pediatric rehabilitation, since they may simultaneously address functional motor deficits and promote early integration into peer micro-communities, cooperative play, and family-centered care [9,10]. Circuit-format training, in which participants progress through a sequence of task-specific stations, offers a structured yet adaptable framework for targeting multiple neuro-motor domains (proprioception, dynamic equilibrium, coordination, and strength) within a single session, while music-assisted delivery has been shown to support rhythmic entrainment and motor timing in populations with neuromotor impairment [11,12]. Despite growing interest in group and circuit-based pediatric protocols, few studies have quantified their effects using a combination of anthropometric (segmental circumference), manual muscle testing, and standardized balance outcomes such as the Berg Balance Scale (BBS) in children with DS specifically [13,14]. Most existing exercise trials in this population report a single outcome domain (typically balance or gross motor function alone), and few combine a music-assisted, group circuit format with simultaneous anthropometric, segmental strength, and balance measurement in the same cohort; this combination, together with a session-by-session circuit structure explicit enough to be replicated, is what the present study adds to the existing literature [6,7,8], rather than a claim of superiority over other exercise modalities. The present study therefore aimed to evaluate the effects of a 12-week, once-weekly, group circuit kinetotherapy program, delivered with musical accompaniment, on segmental muscle trophicity, manual muscle test scores, and functional postural balance in children with trisomy 21, using a baseline (non-repeated) control group for descriptive comparison only.

## 2. Materials and Methods

### 2.1. Study Design

This study was designed as a prospective, exploratory, pre–post intervention study with a non-randomized baseline control group, rather than as a fully controlled comparative trial. Functional parameters were followed longitudinally in the experimental group at two key time points: baseline assessment (T1) and final assessment (T2), performed after completion of the intervention period. The control group underwent the same baseline (T1) evaluation but was not reassessed at T2; no post-intervention data are therefore available for this group, and the design does not permit a formal between-group comparison of change over time. Consequently, improvements observed in the experimental group cannot be fully distinguished from maturation, familiarization with repeated testing, or other concurrent activities occurring over the same 12-week period (see Section 4.6). Information on physical education, sport participation, home exercise, or other rehabilitation therapies received by either group during the study period was not systematically monitored; therefore, “no structured intervention” in the control group indicates only the absence of the study protocol itself, not the absence of all physical activity, and comparable information was not collected for the experimental group either. The interventional protocol was applied exclusively to the experimental group.

### 2.2. Participants

Children were recruited from an outpatient pediatric rehabilitation clinic between February and April 2026, with baseline assessments performed between March and April 2026. Allocation to the experimental or control group followed a convenience (non-random) method: children were assigned to whichever group matched their families’ ability to attend the weekly sessions, rather than by a random-number generator, coin/dice randomization, or matched-pairs procedure. The target sample size (n = 10 per group) was determined by feasibility of recruitment within the study period rather than by an a priori power calculation; with n = 10 per group, the study is likely underpowered to detect small-to-moderate effects, and non-significant results should not be interpreted as evidence of no effect. Both limitations are revisited in Section 4.6. Twenty children diagnosed with trisomy 21, born between 2014 and 2022 and aged 4–12 years at the time of evaluation, were enrolled and allocated to an experimental group (n = 10) and a control group (n = 10). As shown in Table 1, the two groups differed descriptively in age, sex distribution, body mass, and arm circumference; these differences should be taken into account when interpreting the results, and the groups should not be regarded as closely matched. The inclusion criteria were as follows: confirmed diagnosis of Down syndrome (trisomy 21); age between 4 and 12 years at the time of evaluation; independent ambulation (even with a broad support base); sufficient neuro-cognitive capacity to understand and execute simple verbal instructions; and medical clearance from a specialist physician (pediatrician, neurologist, or cardiologist) attesting tolerance to moderate dynamic physical effort. The exclusion criteria were as follows: severe, decompensated congenital cardiac malformations or absolute contraindication to dynamic physical effort; atlanto-axial instability, given the risk of spinal cord compromise during complex movements; acute secondary orthopedic conditions (recent fractures or sprains); major uncorrected sensory impairment; marked oppositional or aggressive behavior that could compromise group dynamics or participant safety; for the experimental group only, a post-inclusion exclusion criterion of absenteeism greater than 20% of scheduled sessions.

### 2.3. Assessment Methods

Functional profiling and monitoring of intervention effects were performed using standardized clinical assessment tools recognized in pediatric rehabilitation. Testing addressed three domains: anthropometric development, gross muscle strength, and static/dynamic postural control. A retrospective review of key neuro-motor developmental milestones was performed for each subject, recording the age (in months) at which independent, unsupported sitting and independent bipedal locomotion were achieved. Associated comorbidities (cardiac, thyroid, or other) were screened, and parental perception of the child’s general muscle tone was recorded.

Anthropometric data were collected to characterize muscle trophicity and somatic status, including body weight (kg) and height (cm). Segmental limb circumferences were measured bilaterally with a tape measure, including relaxed arm circumference; arm circumference during maximal isometric contraction; thigh circumference.

To standardize anthropometric measurements, circumferences were measured with the child in a relaxed standing position at defined anatomical landmarks (mid-point between the acromion and olecranon for arm circumference; mid-point between the inguinal crease and the superior pole of the patella for thigh circumference); three repeated measurements were taken and the mean value was used for analysis. Measurements were obtained using a Gulick tape measure applied with constant light, ensuring contact with the skin without compressing the underlying tissue. All assessments were performed by a single physiotherapist who had received standardized training in the measurement protocol prior to data collection. Assessments were conducted 48 after the last exercise session to minimize the acute effects of exercise on the measurements. Because segmental limb circumference is an indirect and non-specific measure, changes in this parameter cannot, on their own, be equated with muscle hypertrophy; they may also reflect somatic growth, changes in body composition (e.g., subcutaneous adipose tissue), or measurement variability, particularly in a pediatric sample spanning a wide age range. This is considered further in the Section 4.1 and Section 4.6.

Manual muscle testing (MMT) was performed on a functional scale from F1 to F5 for key muscle groups involved in postural control and circuit task execution: shoulder girdle and upper limb—middle deltoid and supraspinatus, elbow flexors (biceps brachii), triceps brachii; pelvic girdle and lower limb—pelvic fixation function, hip flexors (iliopsoas), and knee extensors (quadriceps femoris).

Manual muscle testing followed standard procedures, with the child positioned according to the muscle group being tested (e.g., side-lying or supine for hip flexors/iliopsoas) and manual resistance applied by the examiner against the tested movement, graded on the F1–F5 scale. “Pelvic fixation function” refers operationally to the ability to stabilize the pelvis against examiner-applied resistance during hip flexion/extension testing. Outcome assessors were not blinded to group allocation or to assessment timing (T1 vs. T2); because MMT and BBS scoring involve examiner judgement, this lack of blinding may have introduced observer expectation bias favoring post-intervention scores in the experimental group and is acknowledged as a limitation (Section 4.6). Manual muscle testing followed the grading conventions described in [15]; we are not aware of studies formally validating MMT reliability specifically in children with Down syndrome or comparable intellectual disability, and this is noted as a limitation, since reliability established in typically developing populations may not fully generalize to children with cognitive impairment (Section 4.6).

The central outcome for postural control was the Berg Balance Scale (BBS), a 14-item clinical instrument in which each item is scored from 0 to 4 according to the subject’s independence, stability, and safety in performing the task (maximum total score = 56). The 14 items were as follows: sitting to standing; standing to sitting; transfers; standing unsupported; sitting unsupported with feet on the floor; standing unsupported with eyes closed; standing unsupported with feet together; standing with one foot in front; standing on one leg; turning 360°; turning to look behind over left and right shoulders; retrieving an object from the floor from a standing position; placing alternate foot on a step/stool; and reaching forward with outstretched arm while standing.

The BBS was originally developed and validated in adult populations; a Down syndrome-specific or pediatric-normed minimal clinically important difference (MCID) has not been established, and the present study administered the standard (adult-format) 14-item BBS without formal pediatric adaptation. Accordingly, although the observed mean improvement of approximately 4.5 points was statistically significant, it is not interpreted here as evidence of a clinically meaningful change in the absence of a validated pediatric threshold for this population (see Section 4.3).

The rehabilitation program was applied exclusively to the experimental group over 12 consecutive weeks, at a frequency of one session per week. Each session was delivered in a group format using a music-assisted dynamic circuit training methodology; the musical stimulus served the dual purpose of increasing engagement and compliance and providing an external rhythmic cue for movement coordination. Total session duration was 60 min, structured into a warm-up, the dynamic circuit, and a cool-down phase.

Sessions were delivered in a group of 10 children. Of the total 60-min session, the warm-up lasted approximately 10 min, the circuit approximately 40, with a rest interval of approximately 1–2 min between stations and the cool-down approximately 10 min. The time children spent in active exercise was therefore shorter than the full 60-min session. A once-weekly session frequency was chosen to make attendance feasible for families alongside school and other commitments. This frequency, together with the absence of a formally documented progression scheme, limits conclusions about the optimal exercise dose (see Section 4.6). Each session began with a warm-up track designed to prepare the cardiovascular and respiratory systems and to optimize joint flexibility, beginning with analytic and global joint mobilizations, followed by corrective gait patterns: toe walking with upper limbs maintained in full overhead extension, targeting eccentric loading of the triceps surae and activation of the scapular girdle (deltoid and supraspinatus), and heel walking, targeting activation of the tibialis anterior and re-education of balance through controlled displacement of the center of gravity.

The core of the intervention consisted of a sequence of seven task-specific stations, designed to address axial hypotonia, ligamentous laxity, and dynamic postural control deficits identified at baseline testing. Station 1—Coordination and plyometrics (agility ladder): Participants performed successive floor-plane jumps using a horizontal agility ladder, aimed at developing closed-kinetic-chain explosive strength and improving lower-limb motor coordination. Station 2—Postural re-education and axial alignment (kinematic balance beam): Participants walked in line along a narrow, low therapeutic beam. The biomechanical demand of a reduced base of support required activation of proximal hip and pelvic stabilizers. Station 3—Multidirectional proprioceptive stimulation (irregular stepping stones): This station consisted of a sinuous path of irregular, dome-shaped modules mimicking river stones. The asymmetric arrangement required continuous adaptation of step length and foot placement, targeting ligamentous laxity and calcaneal valgus through direct mechanical stimulation of plantar receptors and reflex activation of the intrinsic foot muscles and tibialis anterior; transitions between convex surfaces also required controlled unipodal weight transfer, activating proximal hip and pelvic stabilizers. Station 4—Spatial orientation and joint flexibility (therapeutic hoop): Participants passed through a large vertical hoop, requiring adaptation of body schema in space and controlled trunk flexion during guided stepping or passage. Station 5—Anticipatory stability and weight transfer (spaced rectangular blocks): Participants stepped over rectangular blocks placed at variable distances, immediately followed by lateral jumps and backward walking, requiring eccentric muscular control and rapid postural reorganization upon abrupt changes in the direction of travel. Station 6—Dynamic postural control and functional reach (unstable surface and target): Children stood on an unstable surface (proprioceptive cushion or Bosu disc) and threw sandbags at a fixed target, integrating reflexive postural stabilization on a mobile surface with voluntary, ballistic upper-limb movement. Station 7—Integrated team activity (ball game): The final station introduced direct cooperation through ball passing within the group. Each reception and pass was paired with a motor task—a squat (knee flexor/extensor control), a lateral throw (trunk rotation), or an overhead throw—to stimulate the strength and axial mobility parameters measured by manual muscle testing. The final phase of each session was accompanied by a slow-tempo track to promote relaxation, using guided breathing techniques and controlled stretching of the major muscle groups engaged during the circuit, aiming to gradually reduce heart rate and post-exercise muscular tension.

### 2.4. Statistical Analysis

Data were centralized in Microsoft Excel and analyzed using Jamovi software v2.7 Distributional normality of continuous outcomes (circumferences, BBS) was not formally tested with a goodness-of-fit test (e.g., Shapiro–Wilk) in the original analysis; given the small sample size (n = 10 per group) and the ordinal nature of the MMT grades, the non-parametric Wilcoxon signed-rank test is reported as the primary within-group test throughout, with the paired Student’s *t*-test retained only as a supplementary reference for the continuous circumference and BBS outcomes. No single pre-specified primary outcome was designated; segmental limb circumferences, MMT grades, and the total BBS score were analyzed as a set of exploratory outcomes, and results should be interpreted accordingly. For each outcome, paired-samples Student’s *t*-test was applied to compare baseline (T1) and final (T2) circumference and BBS measurements within the experimental group; because MMT grades are ordinal, the paired *t*-test is not reported for this outcome, and the non-parametric Wilcoxon signed-rank test is used as the sole inferential test for MMT. The Wilcoxon test was additionally computed for the circumference and BBS outcomes as a distribution-robust cross-check, not as independent confirmation of the parametric result. For MMT, descriptive statistics are reported as median (IQR) rather than mean ± SD, consistent with their ordinal level of measurement, and the frequency distribution of grades at T1 and T2 is reported to allow inspection of paired transitions between grades. Inter-group comparison at baseline was performed using independent-samples Student’s *t*-tests for continuous variables and Fisher’s exact test for the binary sex variable (see Table 1 note) to verify sample homogeneity. No correction for multiple comparisons was applied across the multiple circumference, strength, and balance measures; consequently, all reported *p*-values should be regarded as exploratory (hypothesis-generating) rather than confirmatory, and isolated statistically significant findings should be interpreted with caution given the number of comparisons performed. A *p*-value < 0.05 was considered to indicate nominal statistical significance.

## 3. Results

**Figure 1:**
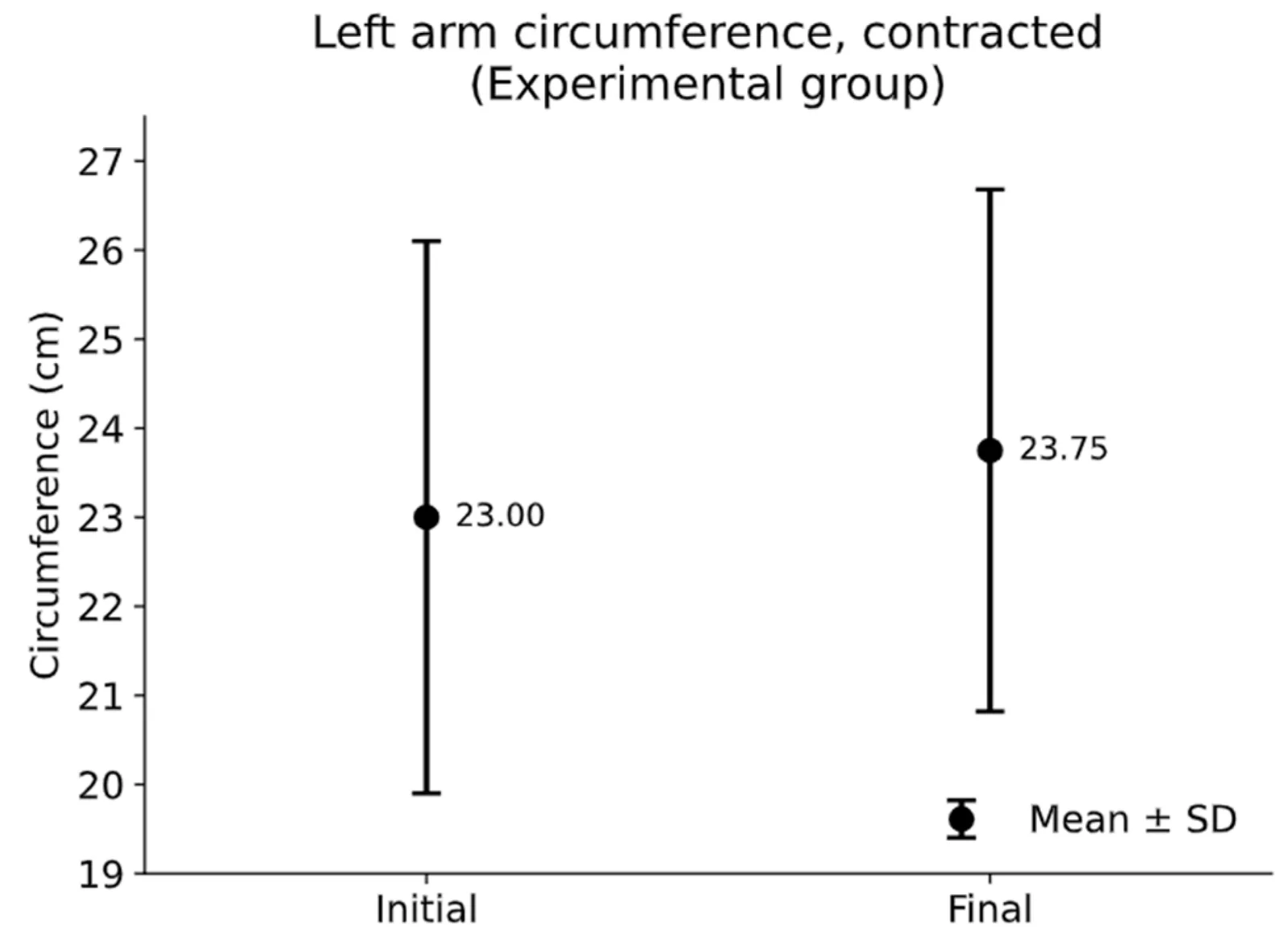
Left arm circumference (contracted), initial T1 vs. final evaluation T2, *p* = 0.009.

**Figure 2:**
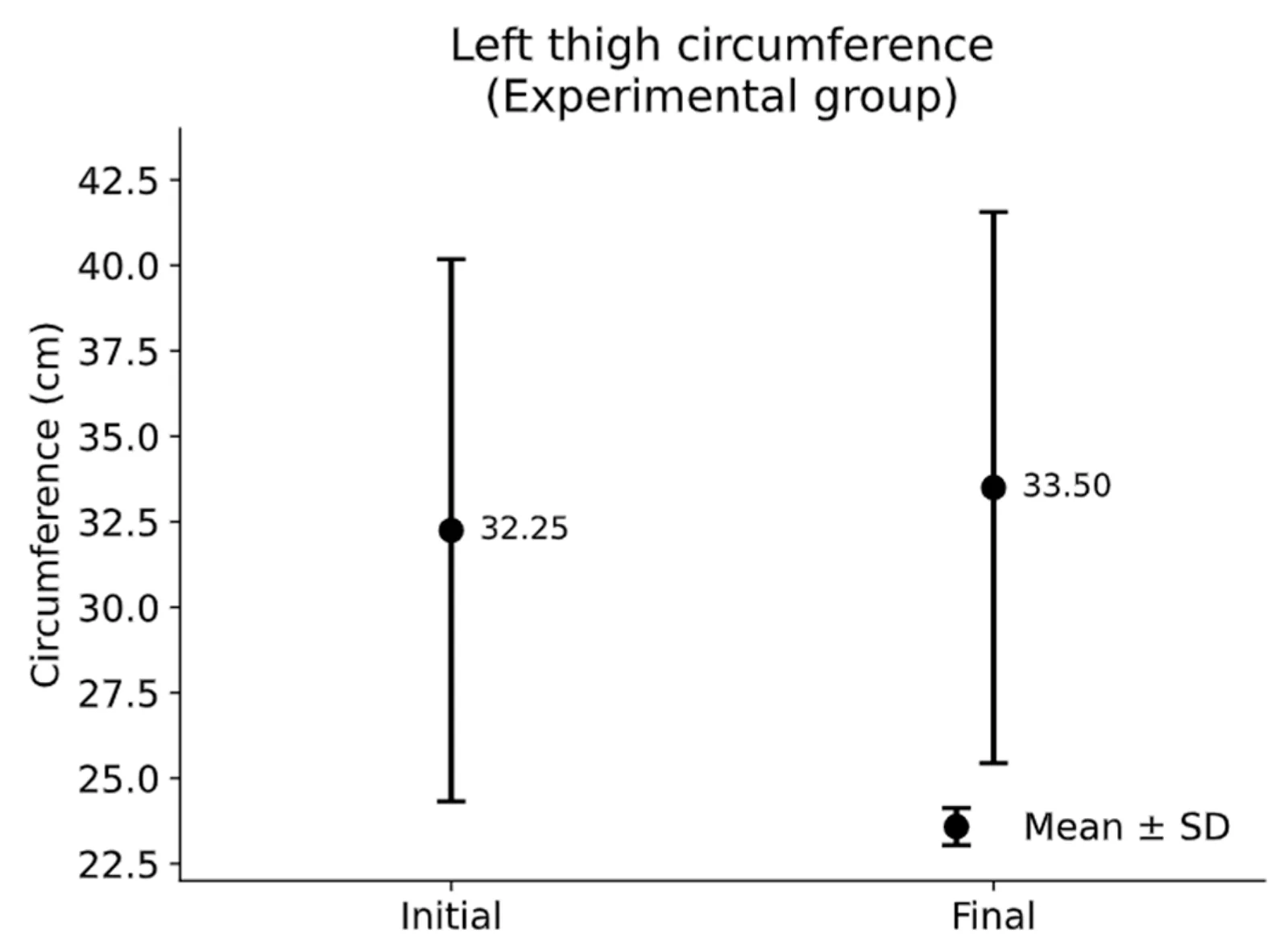
Left thigh circumference, initial T1 vs. final evaluation T2, *p* = 0.001.

**Figure 3:**
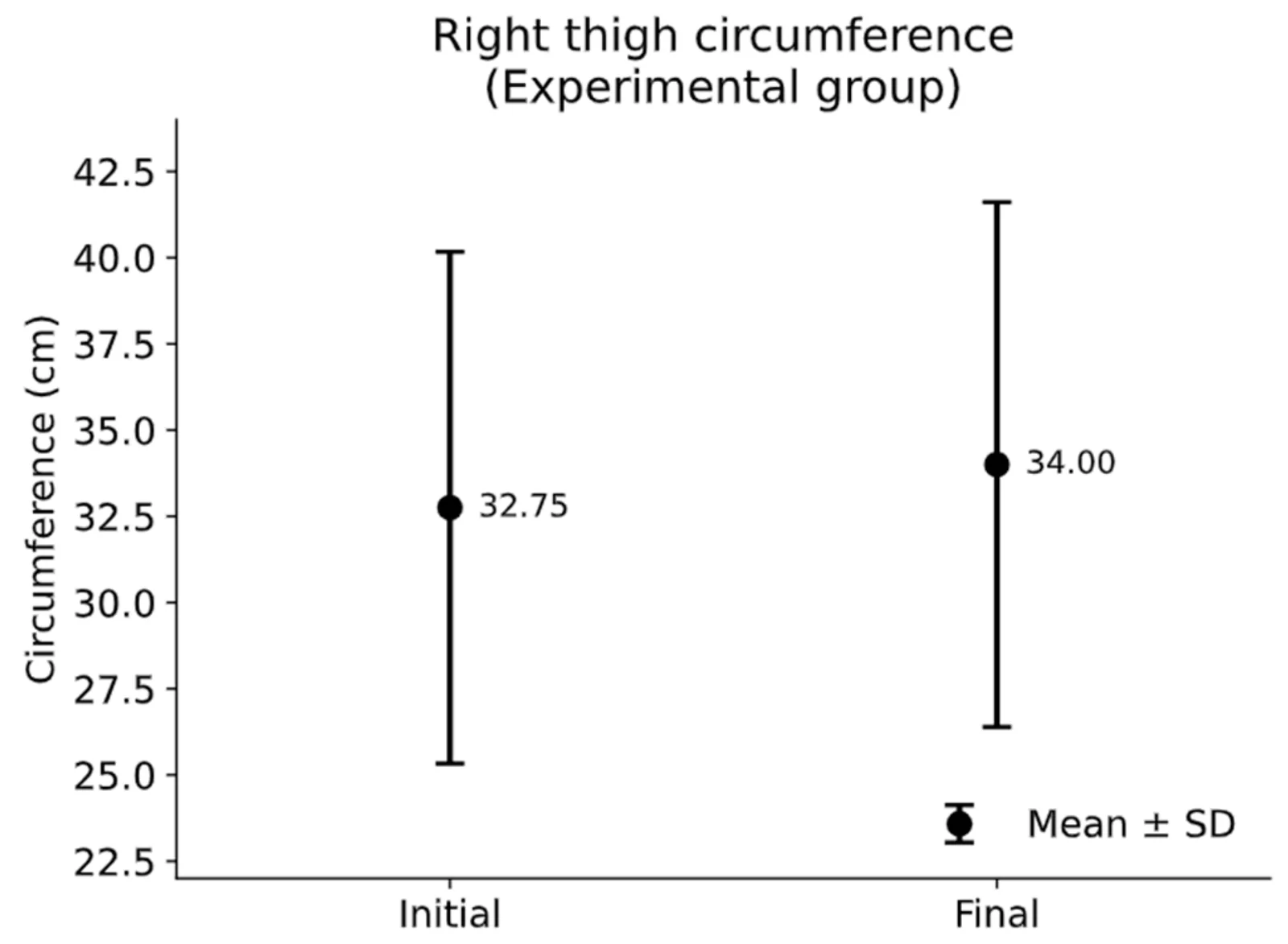
Right thigh circumference, initial T1 vs. final evaluation T2, *p* = 0.003.

Ten children with trisomy 21 were allocated to the group receiving circuit kinetotherapy (experimental group) and ten to the control group. Demographic data, somatometric, and early-development characteristics are summarized in Table 1; as noted above, the groups differed descriptively in age, sex distribution, body mass, and arm circumference. In the experimental group, for each patient the segmental limb circumferences were evaluated as summarized in Table 2. Left arm circumference (contracted) increased significantly over the intervention period (*p* = 0.009, Figure 1); because handedness was not formally recorded, this change cannot be attributed to the dominant versus non-dominant segment and is reported descriptively as a change in left-arm circumference rather than as evidence of a lateralized hypertrophic response. Right arm circumference (contracted) showed a modest upward trend that did not reach significance (*p* = 0.173). Both thigh circumferences increased significantly (left thigh, *p* = 0.001, Figure 2; right thigh, *p* = 0.003, Figure 3). These circumference changes may reflect increased muscle trophicity, but growth, changes in body composition, or measurement variability cannot be excluded as contributing factors, particularly given the wide age range of participants and the absence of post-intervention body-mass and height data (see Section 4.1).

**Figure 4:**
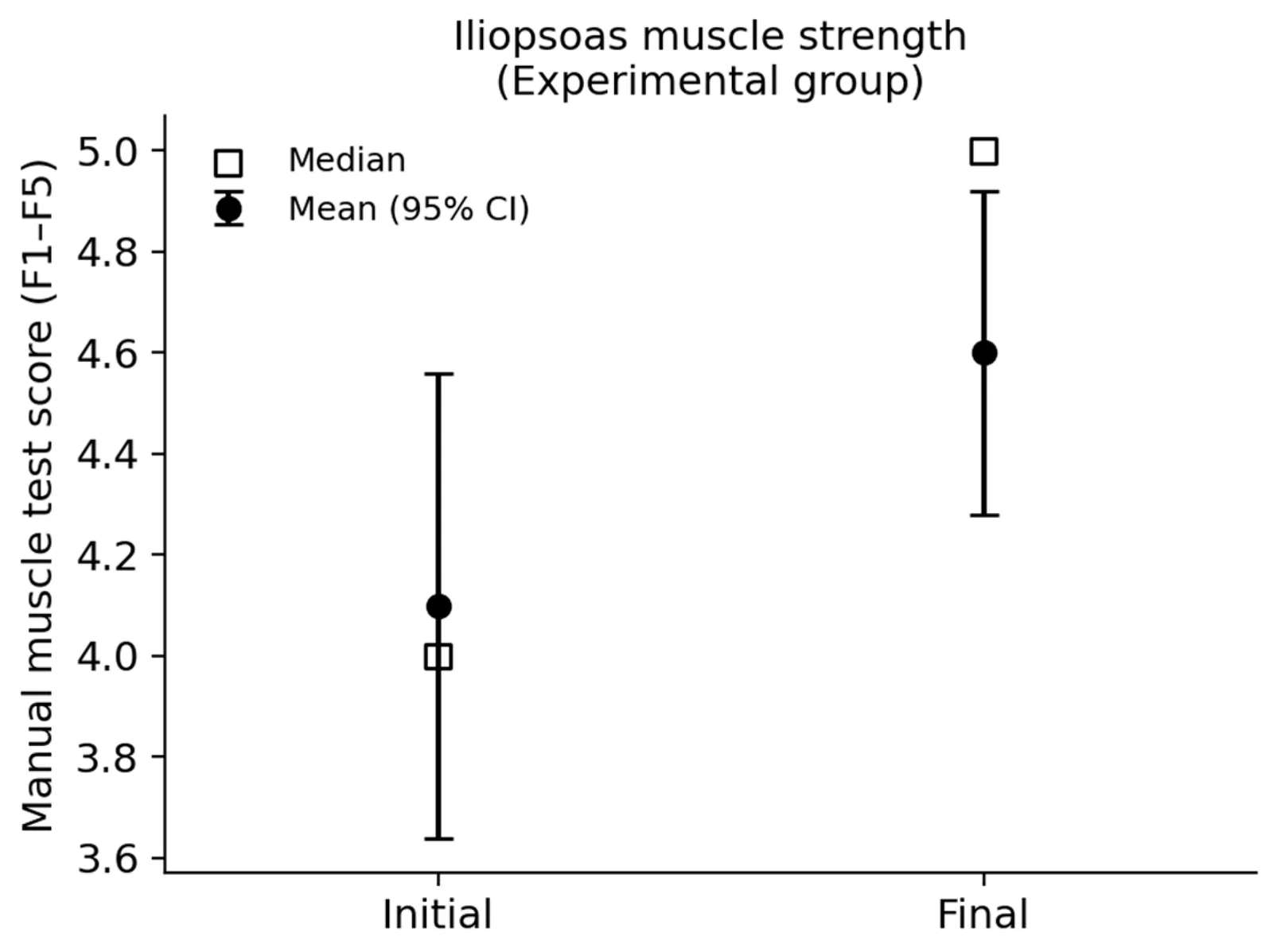
Evolution of iliopsoas manual muscle test scores in the experimental group, initial T1 vs. final evaluation T2, Wilcoxon *p* = 0.015.

Segmental muscle strength was evaluated using manual muscle test (MMT) grades at baseline for both groups and additionally after the intervention for the experimental group; the control group was not reassessed at follow-up. The results are shown in Table 3. Iliopsoas strength was the only parameter to reach the conventional statistical significance threshold (Wilcoxon signed-rank test, *p* = 0.015; the paired *t*-test is not reported for this ordinal outcome, per Section 2.4, Figure 4), consistent with the biomechanical role of this muscle as the principal hip flexor and a key stabilizer of the lumbosacral junction; because no correction for multiple comparisons was applied across the outcomes tested (Section 2.4), this finding should be interpreted as exploratory. Triceps brachii and elbow flexors showed a positive, non-significant trend (*p* = 0.081), whereas the middle deltoid/supraspinatus grade showed a small, non-significant decrease after correction of the baseline value described above (Table 3). Pelvic fixation function and quadriceps femoris, both muscle groups with a predominantly postural/antigravity role, did not reach significance (*p* = 0.346 and *p* = 0.174, respectively). Because the present analyses do not directly test for differences in responsiveness between muscle groups, these patterns are reported descriptively and should not be interpreted as evidence that some muscle groups are inherently more or less responsive to training than others.

**Figure 5:**
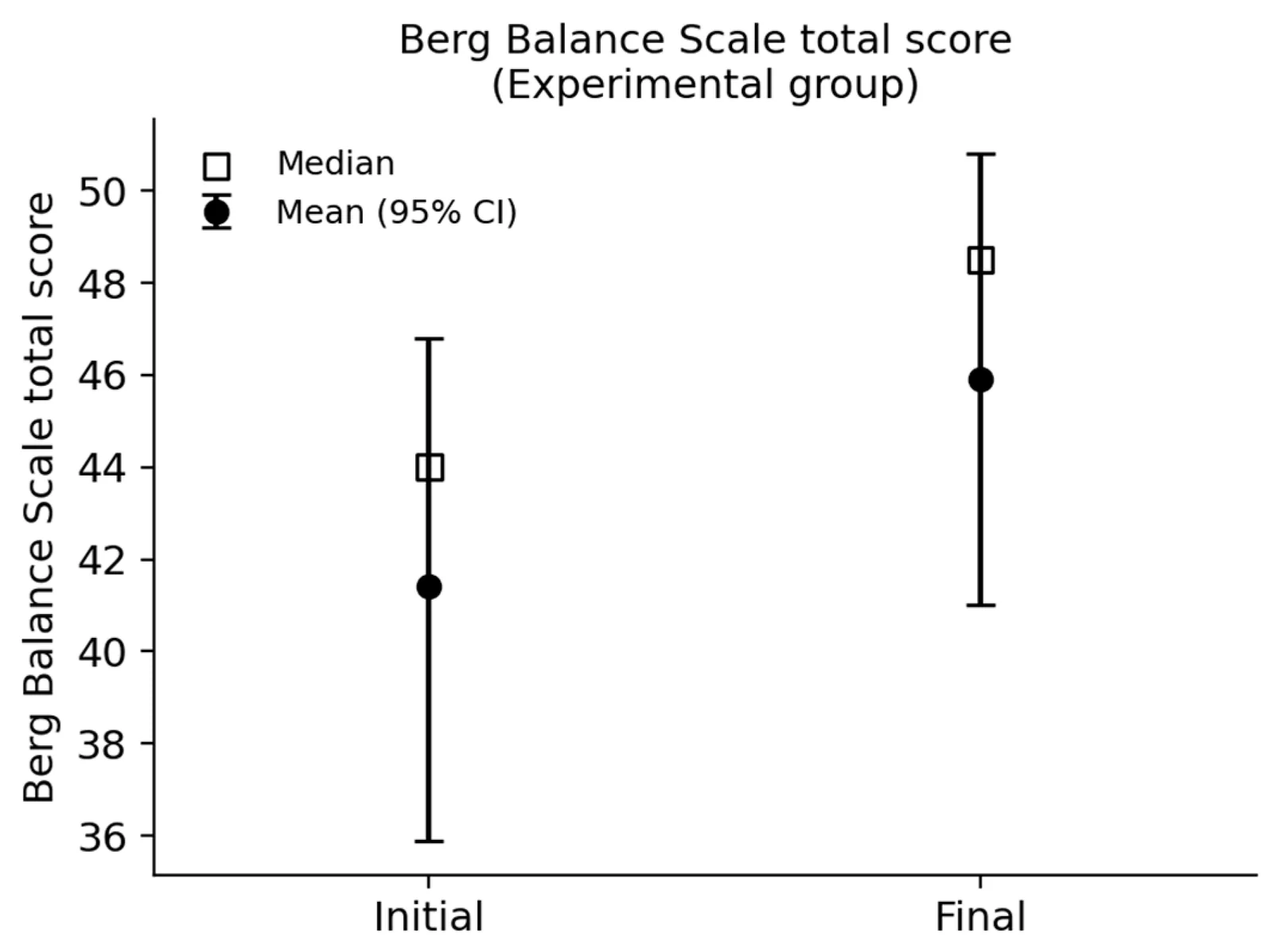
Total Berg Balance Scale score, experimental group, initial T1 vs. final evaluation T2, *p* < 0.001.

The functional balance was assessed using the Berg Balance Scale. The mean BBS score increased from **41.4 ± 8.67** at baseline to **45.9 ± 7.85** after the intervention. This improvement was statistically significant according to both the paired Student’s *t*-test (*p* < 0.001) and the Wilcoxon signed-rank test (*p* = 0.006), Figure 5.

## 4. Discussion

The present study investigated the effects of a structured, group-based circuit kinetotherapy program on muscle trophicity, muscle strength, and functional balance in children with Down syndrome (DS) aged 4–12 years. Overall, the findings indicate a favorable response to the 12-week intervention, particularly with regard to segmental limb circumference, selected muscle groups, and functional balance. The most consistent functional improvement was observed in the Berg Balance Scale (BBS), while the manual muscle testing (MMT) results suggested a more selective pattern of neuromuscular adaptation. These findings are clinically relevant because hypotonia, reduced postural control, ligamentous laxity, altered biomechanics, and delayed motor development are common features of DS and may contribute to limitations in gait, balance, transfers, and participation in daily activities.

At baseline, the two groups were not matched, and the distribution of age and sex was not balanced. Post hoc independent-samples *t*-tests computed from the summary statistics in Table 1 (two-tailed, equal-variance approximation; exact values may differ marginally if recomputed from the raw dataset) indicate that the groups differed significantly in weight (t(18) = 2.37, *p* = 0.029) and in age at independent walking (t(18) = 2.77, *p* = 0.013), showed a non-significant trend toward an age difference (8.50 vs. 6.70 years; t(18) = 1.61, *p* = 0.125), and did not differ significantly in height (*p* = 0.355), age at independent sitting (*p* = 1.000), or sex distribution (Fisher’s exact *p* = 0.37). The experimental group included a greater proportion of older, heavier children, whereas the control group contained a larger proportion of younger, lighter participants. Because motor development and muscle strength naturally change with age and with body size, this baseline imbalance is a critical, uncontrolled confounder: the improvements observed after intervention in the experimental group cannot be attributed to the treatment, and maturation, growth, and regression to the mean must be considered at least as plausible explanations. The pre–post findings in the experimental group should therefore be read as preliminary, hypothesis-generating observations from a single-arm design rather than as evidence of a treatment effect.

### 4.1. Changes in Segmental Muscle Circumference

One of the most notable findings was the change in segmental limb circumference following the intervention. The response was not uniform across anatomical regions, suggesting that the mechanical demands imposed by individual circuit stations may have influenced the pattern of muscular adaptation. The most pronounced changes were observed in the thigh segments, with statistically significant increases in both the left thigh (*p* = 0.001) and right thigh (*p* = 0.003). These findings are particularly relevant because the lower limbs play a central role in weight bearing, gait initiation, postural stabilization, stepping, squatting, and propulsion [16]. The observed increase in thigh circumference may reflect improved muscular trophicity associated with repeated functional loading. Circuit exercises that involve standing, stepping, rising from a seated position, jumping, and other weight-bearing activities repeatedly expose the lower-limb musculature to mechanical demands. In children with DS, such activities may be particularly important because hypotonia and reduced muscle strength can limit the ability to generate and sustain adequate postural forces. Repeated, progressively adapted loading may therefore provide a practical stimulus for improving muscular capacity while simultaneously reinforcing functional motor patterns [6]. The upper-limb response was more heterogeneous, with a significant increase in contracted left-arm circumference (*p* = 0.009) but not in the right arm. Because handedness was not recorded, this asymmetry is reported descriptively and is not attributed to limb dominance; it should be considered exploratory. The greater responsiveness of the lower limbs may instead reflect task specificity, since standing balance, transfers, gait, stepping, and jumping within the circuit repeatedly load the hip and knee extensors and flexors more than the upper limbs [17].

### 4.2. Changes in Muscle Strength

The MMT results provide further evidence of a selective neuromuscular response, which should be interpreted as exploratory given that no correction for multiple comparisons was applied (Section 2.4). Although mean scores increased for most assessed muscle groups, statistical significance was reached only for the iliopsoas. The mean iliopsoas score (median [IQR]) increased from 4.0 [4.0–4.8] at baseline to 5.0 [4.0–5.0] following the intervention (mean ± SD shown in Table 4 for reference). This improvement was supported by the Wilcoxon signed-rank test (*p* = 0.015); consistent with Reviewer 2’s comment that a paired *t*-test is not appropriate for this ordinal outcome, the *t*-test result is no longer reported for MMT. The iliopsoas may have responded particularly well because of its central role in hip flexion and lower-limb advancement during gait and stepping. Hip flexor activity is also important during transitional movements and contributes to the dynamic control of the pelvis and lower extremities. Repeated activation of this muscle group during stepping and other functional exercises may therefore have produced a sufficiently consistent stimulus to result in detectable improvement [18]. The distribution of MMT grades supports this interpretation. At baseline, 20% of participants had an iliopsoas score of F3, 50% had F4, and 30% had F5. Following the intervention, no participant remained at F3, 40% were classified as F4, and 60% achieved F5. Thus, the improvement was not merely a change in the mean score but also reflected a shift toward the highest strength category. Because the present analyses do not directly test for differences in responsiveness between muscle groups, the iliopsoas result should not be interpreted as evidence that this muscle is inherently more trainable than the others; it is reported as the only group crossing the nominal significance threshold in this small, exploratory sample. The other muscle groups showed a more mixed pattern. Triceps brachii increased from 3.90 ± 0.88 to 4.20 ± 0.63, and elbow flexors increased from 4.20 ± 0.79 to 4.50 ± 0.53 (both non-significant, *p* = 0.081). Quadriceps femoris increased from 4.10 ± 0.99 to 4.50 ± 0.53 (*p* = 0.174), and pelvic fixation function increased from 4.20 ± 0.63 to 4.40 ± 0.52 (*p* = 0.346). The middle deltoid/supraspinatus grade, once the baseline mean was corrected to 4.20 ± 0.63 following verification against the reported frequency distribution (see Table 3 notes), showed a small, non-significant decrease to 4.10 ± 0.74 rather than an increase; this pattern should be reported descriptively rather than interpreted as a training effect. These non-significant changes should not be interpreted as evidence that the intervention had no effect, nor should the contrast between the significant iliopsoas finding and the non-significant findings in other muscles be read as proof that these muscle groups respond differently to training, since this was not directly tested. With only 10 participants, statistical power was limited, particularly when baseline scores were already relatively high. A ceiling effect may also have contributed to the lack of statistical significance for some muscle groups. Participants who began with F4 or F5 had less room for measurable improvement than those with lower baseline strength. At baseline, the proportion of participants already at the maximum grade (F5) was 30% for middle deltoid/supraspinatus, 40% for elbow flexors, 20% for triceps brachii, 30% for pelvic fixation function, 30% for iliopsoas, and 40% for quadriceps femoris (Table 3); combined with those already at F4, 80–90% of participants started at F4 or F5 in most of these groups, leaving limited room for further measurable gain. Larger samples, with correction for multiple comparisons, would be necessary to determine whether these observed changes represent reproducible intervention effects.

### 4.3. Functional Balance

The most compelling functional finding was the improvement in BBS total score. The mean score increased from 41.4 ± 8.67 at baseline to 45.9 ± 7.85 following the intervention, representing an absolute mean increase of approximately 4.5 points. The improvement was statistically significant using both the paired Student’s *t*-test (*p* < 0.001) and the Wilcoxon signed-rank test (*p* = 0.006). Statistical significance alone does not establish that this change is clinically meaningful: a Down syndrome-specific or pediatric-normed minimal clinically important difference for the BBS has not been established (see Section 2.3), so this 4.5-point improvement is reported as a statistically significant within-group change rather than as a confirmed clinically meaningful one. Balance is nonetheless a multidimensional motor function requiring integration of muscle strength, postural control, proprioception, anticipatory adjustments, and the ability to adapt the center of mass during static and dynamic tasks, and children with DS frequently experience difficulties in these domains; the observed increase in BBS score is consistent with, but does not by itself prove, an effect of the intervention on the coordination of the multiple systems (muscle strength, postural control, proprioception, anticipatory adjustment) that balance requires [19,20]. Given the single-arm, non-randomized design, this association should be read as a hypothesis for future controlled testing rather than as evidence that the intervention caused the change. The circuit format may have contributed to this improvement by repeatedly exposing participants to varied balance challenges. Changing body positions, shifting the center of gravity, stepping, transferring weight between limbs, and maintaining stability during movement require continuous adjustment of postural strategies. Repetition of these activities within a structured environment may facilitate motor learning and improve confidence during functional tasks [20]; these gains in BBS test performance should be distinguished from demonstrated transfer to daily activities, school participation, or sport, which were not assessed in this study. The improvement in BBS should, however, be interpreted as a within-group effect. Because post-intervention BBS data were unavailable for the control group, it is not possible to establish that the improvement was greater than any change that might have occurred naturally over the same period in untreated participants. Consequently, the current findings provide evidence of improvement following the intervention but do not constitute definitive evidence of superiority over usual activity or standard care.

### 4.4. Relationship Between Proximal Stability and Functional Performance

An important clinical consideration emerging from the results is the potential role of proximal stability in improving global postural control. The pelvic fixation function showed an increase in mean MMT score from 4.20 ± 0.63 to 4.40 ± 0.52. Although this change did not reach statistical significance, it occurred alongside the significant improvement in BBS performance; however, the present study did not directly test an association between pelvic fixation function and BBS score, so this co-occurrence should not be read as evidence that one caused or contributed to the other. From a biomechanical perspective, adequate pelvic control provides a stable foundation for movement of the trunk and lower extremities, and improved control of the pelvis may facilitate weight transfer, stepping, gait initiation, and maintenance of the center of mass within the base of support. This is presented as a plausible hypothesis—that targeted strengthening of proximal musculature could contribute to improvements in global balance—rather than as a mechanism established by the present measurements; testing the association between pelvic fixation function and BBS score directly would be needed to confirm it. With this caveat, the findings are consistent with a rehabilitation strategy that does not focus exclusively on isolated strengthening but integrates strengthening with functional, weight-bearing, and balance-oriented activities. Such an approach may be particularly relevant for children with DS because improvements in muscle strength are most valuable when they translate into better functional movement and participation [21,22].

### 4.5. Clinical Relevance of the Circuit-Based Approach

The circuit organization of the intervention represents another important aspect of the study. Unlike isolated therapeutic exercises, circuit kinetotherapy allows multiple motor components to be trained sequentially within a single session. This structure can incorporate strengthening, balance, coordination, weight shifting, gait-related activities, and functional transitions while allowing the therapist to adjust the difficulty according to the child’s abilities. The group-based format may provide additional advantages. Children perform activities in a socially interactive environment, which may increase motivation and engagement. Musical accompaniment may further contribute to rhythm, attention, and task organization. Because the program combined exercise, music, and group interaction simultaneously, their individual contributions cannot be separated with the present design, and claims about motivation, compliance, or additional benefits attributable specifically to the musical component should be regarded as qualified hypotheses rather than established effects, particularly because adherence, motivation, and engagement were not formally measured. Although adherence and motivation were not formally measured in the present study, the practical feasibility of a group circuit format suggests that this approach may be suitable for pediatric rehabilitation settings where individualized therapy time and resources are limited. These observations are broadly consistent with previous reports suggesting that structured physical activity and exercise can improve balance, motor performance, and functional outcomes in children with DS [23]. The present findings add to this literature by suggesting that a circuit-based approach combining therapist-adjusted task difficulty (rather than a formally documented progressive-resistance protocol; see Section 2.3 and Section 4.6), functional movement, balance challenges, and group participation may produce measurable changes within a relatively short intervention period [4].

### 4.6. Limitations and Future Directions

Most fundamentally, because the control group was not reassessed at T2, this study is, in design terms, a single-arm pre–post (within-subject) study in the experimental group with a baseline-only reference group, not a true two-arm comparative or controlled trial; this severely limits causal inference, and none of the findings above should be read as evidence that the intervention, rather than maturation, testing familiarity, or concurrent activity, produced the observed changes. Several limitations should be considered when interpreting these findings. First, the sample was small, with only 10 participants per group, substantially limiting statistical power and increasing the influence of individual variability. This is particularly relevant for the outcomes that showed favorable trends but did not reach statistical significance. Second, the age and sex distributions were not fully matched between groups. The experimental group contained older children, whereas the control group included more younger participants. Since strength, balance, and motor competence develop with age, maturation may have influenced the results. Third, post-intervention measurements were unavailable for the control group for several outcomes. Consequently, definitive between-group comparisons could not be performed. Future studies should prioritize complete follow-up assessment in both groups. Fourth, the intervention was conducted over 12 weeks with sessions once weekly. This design does not permit conclusions regarding dose–response relationships, optimal frequency, or long-term retention. Future trials should compare different intervention frequencies and include follow-up assessments several months after treatment. Fifth, handedness was not documented, which limits interpretation of the asymmetric upper-limb circumference findings. Standardized assessment of limb dominance should therefore be incorporated into future protocols. Sixth, allocation to groups was not randomized but based on feasibility of attendance, and the target sample size was likewise set by feasibility rather than by an a priori power calculation; both are acknowledged sources of potential selection bias and imprecision. Seventh, attendance, withdrawals, exclusions under the >20% absenteeism criterion, reasons for missing assessments, and adverse events or session modifications were not comprehensively reported in the original submission (see Section 2.2); this limits assessment of the program’s feasibility and of possible selection effects among completers. Eighth, physical education, sport participation, home exercise, and other rehabilitation received by either group during the study period were not systematically monitored, so activities outside the study protocol cannot be excluded as contributors to the observed changes. Ninth, a structured, criterion-based progression scheme for task difficulty, resistance, or volume was not implemented or formally recorded; the intervention is therefore more accurately described as involving therapist-adjusted task difficulty than a documented progressive-resistance protocol. Tenth, the Berg Balance Scale was administered in its standard (adult-validated) form without a Down-syndrome-specific or pediatric-normed minimal clinically important difference, limiting claims of clinical (as opposed to statistical) meaningfulness. Eleventh, no correction for multiple comparisons was applied across the several circumference, strength, and balance outcomes tested, so all reported *p*-values should be regarded as exploratory. Twelfth, a data-verification exercise prompted by peer review identified and corrected an internal inconsistency in the originally reported baseline mean for middle deltoid/supraspinatus MMT scores (Table 3); this is now reported accurately, and readers should note that this muscle group showed a decrease rather than an increase over the intervention period. Finally, the single-center design and lack of blinded outcome assessment may have introduced selection and measurement bias. Larger multicenter randomized controlled trials with blinded assessors, matched control groups, standardized intervention dosage, and longer follow-up would provide stronger evidence regarding the effectiveness and sustainability of circuit kinetotherapy in children with DS.

## 5. Conclusions

The present study provides preliminary evidence that a structured, group-based circuit kinetotherapy program may positively influence muscle trophicity, selected muscle strength, and functional balance in children with Down syndrome. The most consistent somatometric response was observed in the lower limbs, with significant increases in right and left thigh circumference, suggesting that repeated weight-bearing and functional activities may provide an effective stimulus for lower-limb muscular adaptation. An improvement was also observed in left-arm circumference, although the asymmetric upper-limb findings require cautious interpretation because limb dominance was not assessed. Manual muscle testing demonstrated a selective neuromuscular response. The iliopsoas showed a statistically significant improvement, with a shift toward the highest MMT grade following the intervention. Other assessed muscle groups (triceps brachii, elbow flexors, pelvic fixation function, and quadriceps femoris) demonstrated positive changes in mean strength scores but did not reach statistical significance, potentially reflecting the limited statistical power associated with the small sample. The middle deltoid/supraspinatus grade, once an internal inconsistency in the reported baseline mean, was identified and corrected during peer review and showed a small, non-significant decrease rather than an increase (see Section 3, Section 4 and Section 4.2); because no correction for multiple comparisons was applied, all of these findings, including the significant iliopsoas result above, should be regarded as exploratory. The strongest functional finding was the significant improvement in Berg Balance Scale total score. This finding suggests that circuit-based kinetotherapy may improve functional balance and postural control in children with DS. The combination of proximal stabilization, weight-bearing activities, stepping, transfers, and progressively challenging balance tasks may represent an effective therapeutic strategy for addressing the complex motor deficits associated with DS. However, because post-intervention data were unavailable for the control group, the findings should be interpreted as evidence of within-group improvement rather than definitive proof of treatment superiority. The unequal age and sex distributions and the small sample size further limit the generalizability of the results. Overall, within the constraints of this small, exploratory, non-randomized study, circuit kinetotherapy appears to be a potentially promising adjunct to pediatric rehabilitation for children with Down syndrome; a firm conclusion about feasibility should await systematic reporting of attendance, adverse events, and session modifications (Section 4.6), particularly when exercises are adapted to each child’s ability level (task difficulty adjusted by the therapist), functionally oriented, and delivered in an engaging group environment. Larger randomized controlled studies with adequately matched control groups, blinded outcome assessment, standardized intervention dosage, and longer follow-up are required to confirm these preliminary findings and establish the optimal frequency, duration, and long-term effectiveness of this intervention.

## Figures and Tables

**Table 1:** Demographic, somatometric, and early developmental characteristics at baseline.

	Experimental Group (Circuit Kinetotherapy) (n = 10)	Control Group (n = 10)
**Sex (F/M)**	7/3	4/6
**Age (years)**	8.50 ± 2.55	6.70 ± 2.45
**Height (cm)**	128 ± 18.07	121 ± 14.71
**Weight (kg)**	33.00 ± 12.47	22.50 ± 6.41
**Independent walking (months)**	21.00 ± 4.35	16.50 ± 2.75
**Independent sitting (months)**	8.50 ± 1.71	8.50 ± 1.50

The two groups were not matched. Post hoc independent-samples *t*-tests (or Fisher’s exact test for sex) computed from these summary statistics show a significant between-group difference in weight (*p* = 0.029) and age at independent walking (*p* = 0.013), a non-significant trend for age (*p* = 0.125), and no significant difference in height (*p* = 0.355), age at independent sitting (*p* = 1.000), or sex distribution (*p* = 0.37); see Section 2.2 and Section 4.

**Table 2:** Segmental limb circumferences at baseline (T1, both groups) and after the intervention (T2, experimental group only; not assessed in the control group).

Measurement	Experimental Group (n = 10)	Control Group (n = 10)
**Left arm, relaxed (cm)**		
Initial	22.50 ± 3.28	15.50 ± 1.94
Final	23 ± 3.23	NA
**Left arm, contracted (cm)**		
Initial	23 ± 3.10	16.00 ± 1.98
Final	23.75 ± 2.93	NA
**Right arm, relaxed (cm)**		
Initial	23.25 ± 3.41	16.25 ± 2.00
Final	23.25 ± 3.34	NA
**Right arm, contracted (cm)**		
Initial	23.50 ± 3.27	16.75 ± 2.06
Final	23.75 ± 3.46	NA
**Right thigh (cm)**		
Initial	32.75 ± 7.42	30.25 ± 4.83
Final	34.00 ± 7.61	NA
**Left thigh (cm)**		
Initial	32.25 ± 7.93	29.75 ± 4.82
Final	33.50 ± 8.06	NA

**Table 3:** Distribution and descriptive statistics of manual muscle test (MMT) scores at baseline and after the intervention.

Anatomical Region/Muscle	MMT Grade	Control Group Initial T1 (n = 10)	Experimental Group Initial T1 (n = 10) (%) Mean ± SD	Experimental Group Final T2 (n = 10) (%) Mean ± SD
**Middle deltoid/supraspinatus**	F3/F4/F5	0/2/8	1 (10%)/6 (60%)/3 (30%)	2 (20%)/5 (50%)/3 (30%)
			**4.20 ± 0.63**	**4.10 ± 0.74**
**Elbow flexors**	F3/F4/F5	1/2/7	2 (20%)/4 (40%)/4 (40%)	0 (0%)/5 (50%)/5 (50%)
			**4.20 ± 0.79**	**4.50 ± 0.53**
**Triceps brachii**	F2/F3/F4/F5	0/1/5/4	1 (10%)/1 (10%)/6 (60%)/2 (20%)	0 (0%)/1 (10%)/6 (60%)/3 (30%)
			**3.90 ± 0.88**	**4.20 ± 0.63**
**Pelvic fixation function**	F3/F4/F5	0/2/8	1 (10%)/6 (60%)/3 (30%)	0 (0%)/6 (60%)/4 (40%)
			**4.20 ± 0.63**	**4.40 ± 0.52**
**Iliopsoas**	F3/F4/F5	1/4/5	2 (20%)/5 (50%)/3 (30%)	0 (0%)/4 (40%)/6 (60%)
			**4.10 ± 0.74**	**4.60 ± 0.52**
**Quadriceps femoris**	F2/F3/F4/F5	0/2/2/6	1 (10%)/1 (10%)/4 (40%)/4 (40%)	0 (0%)/0 (0%)/5 (50%)/5 (50%)
			**4.10 ± 0.99**	**4.50 ± 0.53**

MMT, manual muscle testing; SD, standard deviation. MMT grades are expressed as frequencies and percentages for the experimental group. Descriptive statistics are presented as mean ± SD. The control group was assessed only at the initial evaluation. Percentages are calculated within the experimental group (*n* = 10). Higher MMT grades indicate greater muscle strength.

**Table 4:** Consolidated summary of all assessed outcomes, paired within-group changes, and statistical tests in the experimental group (n = 10).

Outcome (Experimental Group)	T1 (n = 10)	T2 (n = 10)	Change	Test Used	*p*-Value	Effect Size
Left arm circumference, contracted (cm)	23.00 ± 3.10	23.75 ± 2.93	+0.75	Paired t	0.009	d = 0.25
Right arm circumference, contracted (cm)	23.50 ± 3.27	23.75 ± 3.46	+0.25	Paired t	0.173	d = 0.07
Left thigh circumference (cm)	32.25 ± 7.93	33.50 ± 8.06	+1.25	Paired t	0.001	d = 0.16
Right thigh circumference (cm)	32.75 ± 7.42	34.00 ± 7.61	+1.25	Paired t	0.003	d = 0.17
Middle deltoid/supraspinatus (MMT)	4.0 (4.0–4.8)	4.0 (4.0–4.8)	0	Wilcoxon	NS	N/A (ordinal)
Elbow flexors (MMT)	4.0 (4.0–5.0)	4.5 (4.0–5.0)	+0.5	Wilcoxon	0.081	N/A (ordinal)
Triceps brachii (MMT)	4.0 (4.0–4.0)	4.0 (4.0–4.8)	0	Wilcoxon	0.081	N/A (ordinal)
Pelvic fixation function (MMT)	4.0 (4.0–4.8)	4.0 (4.0–5.0)	0	Wilcoxon	0.346	N/A (ordinal)
Iliopsoas (MMT)	4.0 (4.0–4.8)	5.0 (4.0–5.0)	+1.0	Wilcoxon	0.015	N/A (ordinal)
Quadriceps femoris (MMT)	4.0 (4.0–5.0)	4.5 (4.0–5.0)	+0.5	Wilcoxon	0.174	N/A (ordinal)
Berg Balance Scale, total score	41.4 ± 8.67	45.9 ± 7.85	+4.5	Paired t(9) = 6.40/Wilcoxon	<0.001/0.006	d = 0.54

MMT rows now report median (IQR) rather than mean ± SD, since MMT grades are an ordinal scale for which a mean is not strictly appropriate; the paired Student’s *t*-test has been removed for these rows and only the Wilcoxon signed-rank test is reported. An approximate Cohen’s d (using the pooled T1/T2 SD as a simple approximation, not a paired-difference SD) is now reported for the continuous circumference and BBS outcomes; it is not reported for MMT, which is ordinal. NS, not statistically significant (grade decreased). No correction for multiple comparisons was applied; all *p*-values are exploratory. Paired 95% confidence intervals for the mean/median differences could not be computed from the summary statistics available and would require the underlying per-participant dataset; individual paired observations are not plotted here for the same reason.

## Data Availability

The original contributions presented in this study are included in the article. Further inquiries can be directed to the corresponding authors.
